# Supplementary material for: Response of Bolivian gray titi monkeys (Plecturocebus donacophilus) to an anthropogenic noise gradient: behavioral and hormonal correlates
Source: PeerJ. 2020 Nov 20;8:e10417. doi: 10.7717/peerj.10417 (PMC7682439; doi:10.7717/peerj.10417)
Supplement: Article S1 [file peerj-08-10417-s001.pdf]

Response of Bolivian gray titi monkeys, *Plecturocebus donacophilus*, to a mannequin: field experiments.

GROUP 1 – MFJ

Trial # 6
Date: 05/7/18
Start time: 5:50
End time: 7:50
Test duration: 3 min

7:45–7:47 Adult male appears ~4 m above ground, stops and looks at the mannequin. He does not give any call, nor exhibit any defensive posture. Then, the adult female appears next to her mate and observes the dummy, with no obvious reaction. Both individuals observe the mannequin for ~5 s and move on. Finally, the juvenile appears and follows the adults, completely ignoring the dummy.

GROUP 2 – MFSJI

Trial # 3
Date: 02/17/18
Start time: 5:45; no wind, cloudy
End time: 6:13
Test duration: 3 min

6:10–6:13 Infant first appears ~2 m above ground, approaching the mannequin to a distance of ~1.5 m. He looks at it briefly and moves away. Then, the juvenile shows up in the same spot where the infant was located. He does not call and continues his path. The adult male emerges, stops and takes a defensive posture, hunches his back and shows piloerection while looking at the dummy and then moves on. Follows the sub-adult male who stops at a distance of ~1.5 m from the dummy and observes for ~30 s, urinates, looks back at the dummy and emits a high-pitched call of short duration (~3 s) before moving on. Finally, appears the adult female who displays

piloerection while looking at the mannequin. She emits a short alarm call (~3 s) and then leaves.

GROUP 3 – MFJIB

Trial # 2
Date: 02/15/18
Start time: 6:00; no wind, sunny
End time: 7:20
Mannequin: north facing
Test duration: 20 min

6:59–7:00 Adult female is the first to observe the mannequin from a vantage point in a Eucalyptus tree, at a distance of ~4 m and ~5 m above ground. She starts emitting soft, high-pitched calls while watching the dummy. The juvenile and infant appear together and observe the dummy from behind the branches. Then, appears the adult male and they all start emitting chirrups. They all climb up to the tree top (~5 m). Then the juvenile starts producing chirrups.

7:01–7:04 Focal group remains silent while watching the mannequin. The adult female goes down and looks directly at the dummy. The adult male stands behind her mate who adopts a defensive posture, shrinking her back and ruffling her coat, staggering from side to side without calling. They do not leap to another tree.

7:05–7:08 Adult female approaches the mannequin to a distance of ~4 m. She stands ~2 m above ground. She is hiding among the branches of a Eucalyptus tree and adopts a defensive posture, hunching her back with signs of piloerection. The adult male starts producing solo song in response to the duet of a neighboring pair. They don't move to another tree.

7:08–7:10 Adult female approaches and inspects the dummy ~3 m away. She stands ~2 m above ground. The juvenile arrives, followed by the two infants. The adult male shows up last and approaches the dummy with a defensive posture. They all walk away and climb in the Eucalyptus trees, behind. At 7:10, the adult male starts

producing solo song for approximately 15 s. Finally, they all climb up to the canopy and move back, avoiding the decoy before crossing to another tree.

GROUP 4 – MFJI

Trial # 1
Date: 02/16/18
Mannequin, north facing
Start time: 5:45; no wind, sunny
End time: 8:20
Test duration: 30 min

7:51–7:55 Adult pair appears ~5 m above ground, in a tree just in front of the mannequin. They emit short high-pitched alarm calls for about 5 s. The infant goes down on a branch ~2 m above ground, right in front of the dummy, without calling, as if he had not seen it yet.

8:00–8:08 The infant allopreens while taking the sun on the branch, then moves to another tree and passes next to the dummy, ignoring it completely. He then starts eating leaves. There is no other individual around.

8:08–8:14 Adult female appears and sees the mannequin but ignores it. She goes behind the infant while circumnavigating the dummy. At a height of ~4 m, just when she is behind the dummy, she emits a short alarm call. As a result, the infant runs away and disappears among the tangles of lianas. The adult male is not yet visible.

8:14–8:21 Adult female looks at the mannequin from between the branches of the trees at a distance of ~5 m and ~4 m high. However, she does not emit any call and start eating leaves. All individuals regroup to forage on leaves. Finally, the adult male stops eating and moves towards the mannequin, followed by the female and the infant. All individuals pass the dummy at a distance of ~2 m and at a height of ~3 m. None of them seems to be scared or intimidated.

GROUP 5 – MFJI

Trial # 4
Date: 04/05/18
Mannequin
Start time: 05:50
End time: 8:15
Test duration: 25 min

7:50–8:00 Adult female appears ~3 m above ground and ~4 m away from the mannequin. She exhibits a defensive posture, arching her back with piloerection, lowers her head and stares at the dummy. She moves her head from side to side and emits an alarm call. She then moves backwards, wobbling again from side to side while watching the mannequin. She emits a second alarm call and joins the group chorus. All individuals are in the canopy, at a height of ~10 m.

8:00–8:15 Infant appears ~2 m above ground and approaches the mannequin, which is ~3 m away. He begins to inspect it, turn its head from side to side and stares at it. Without producing any call, he starts moving up and down the tree while looking at the dummy. He pauses, sits down and keeps looking at the dummy. Then, he starts eating leaves. Both the adult female and the infant climb to the canopy and start foraging with other group members. Finally, they move in the opposite direction and cross the path about 20 m away from the mannequin.

GROUP 6 – MFSJIB

Trial # 1
Date: 04/04/18
Start time: 05:50
End time: 7:00
Test duration: 3 min

The mannequin was put under the sleeping tree of the group, which is ~6 m above ground. At 06:58 an alarm call was heard but the caller could not be identified due to

height and obstructing foliage. Then, all individuals descend at a height of ~5 m and move in the opposite direction to the dummy and finally cross the path. Only the adult male stops briefly (5 s) to observe the dummy in a defensive position, arching his back and showing piloerection.
