# Supplementary material for: Response of Bolivian gray titi monkeys (Plecturocebus donacophilus) to an anthropogenic noise gradient: behavioral and hormonal correlates
Source: PeerJ. 2020 Nov 20;8:e10417. doi: 10.7717/peerj.10417 (PMC7682439; doi:10.7717/peerj.10417)
Supplement: Article S2 [file peerj-08-10417-s002.pdf]

Enzyme-immunoassay of fecal cortisol in Bolivian gray titi monkeys, *Plecturocebus donacophilus*. Parallelism test and validation.

Amélie Chimènes and Audrey Maille

Unité Eco-anthropologie UMR 7206, Museum National d'Histoire Naturelle, CNRS, Université de Paris, Paris, France

1. Reception of the fecal samples of titis (December 31, 2018).

Dr. Patrice Adret, Research Associate at the Museo de Historia Natural Noel Kempff Mercado de Sante Cruz de la Sierra, brought us 30 fecal samples (0.1g each) kept inside 15ml polypropylene tubes in an aqueous solution of 2.5 ml of distilled water and 2.5ml of 96° ethanol. The fresh samples were collected between 03/11/2017 and 05/04/2018 and came from 19 individuals, both adults and young, distributed in 6 family groups. The samples had been kept in a freezer until they were exported to France by Dr. Adret himself who carried them along in a polystyrene container with ice packs, together with proper documentation (Figure 1).

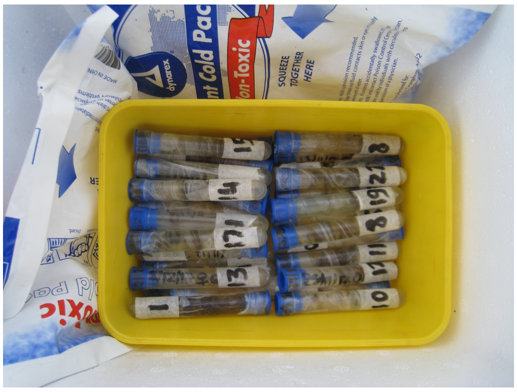

Figure 1. Samples ready for exportation to France.

The meeting took place with Dr. Audrey Maille at the Musée de l'Homme in Paris, France. Subsequently, the tests were carried out by Ms. Amélie Chimènes, Assistant Engineer at the **Palaeogenomics and Molecular Genetics laboratory** of the UMR 7206 directed by Professor Evelyne Heyer.

2. Extraction and drying (January 7 and 8, 2019).

To start with, the contents of the tubes were transferred into 50ml polypropylene tubes to which was added 10ml of absolute ethanol to reach the minimum of 80% alcohol necessary for the complete extraction of fecal cortisol [1-2]. After having vortexed the mixture for 1 minute, the samples remained 4 hours at rest to carry out the extraction. At the end of these 4 hours, 2 ml of supernatant were collected in a 2 ml Eppendorf tube and were stored at 4°C. The following day, 0.5 ml of extract was transferred to a 1.5 ml Eppendorf tube and placed in a desiccator (Concentrator plus by Eppendorf, Figure 2). The drying lasted 4 hours at 1400 rpm. The tubes were then stored at 4°C.

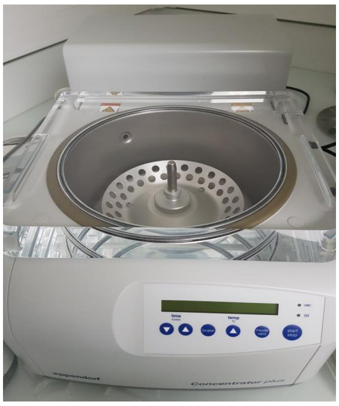

Figure 2. Desiccator used for drying the samples

3. Measurement of fecal cortisol using an EIA assay

The awarded ELISA kit sent by post from the USA was unusable after the customs blocked delivery for too long because storage for one month at room temperature invalidates the quality of the reagents. Therefore, by mutual agreement between Dr. Adret and Dr. Maille, it was agreed to use the salivary cortisol assay kits from Salimetrics © (Lot 1701514) purchased by the Paleogenomics platform for Dr. Maille and which was used for detection of cortisol in feces of bonobos, chimpanzees, orangutans and baboons in another research project (article in preparation).

The antibody used in this IEA kit from Salimetrics © has a high affinity for cortisol (< 0.007 µg/dL) and shows little cross-reactivity with dexamethasone (19.2%), prednisolone (0.57%), corticosterone (0.21%), 11-deoxycortisol (0.16%) and other steroid components (less than 0.15%). Sensitivity of the assay is 0.028 µg/dL (range: 0.012-3.000 µg/dL). Control tests had to be carried out to validate the use of this EIA assay for cortisol measurement in the fecal samples of titis (*Plecturocebus donacophilus)*.

a) First assay: Parallelism test on four fecal samples (January 9, 2019)

In an EIA (Enzyme Immuno Assay), the parallelism test allows both to choose the dilution rate necessary for the determination of a biological element according to the starting material and to ensure the proper functioning of the kit dosing.

Methods for the parallelism test

The complete protocol is visible in appendix 1. It consists of several phases:

– Rehydration of the dried samples with 0.5 ml of test buffer provided in the kit.

– Dilution of 4 samples chosen according to diversity criteria (weight, sex, age, dominance...) with each sample coming from a different group.

– Blanking, standards, controls and samples (in duplicates: Table 1)

Table 1: Plate layout for the blanck (A1 and A2) standards (B1:G1 and B2:G2), controls (H1, H2, A3, A4), and 4 samples used for the parallelism test (B3:H3, B4:H4, A5:H5, A6:H6, A7:E7, A8:E8)

	1	2	3	4	5	6	7	8	9	10	11	12
A	Blanc	Blanc	Ctrl L	Ctrl L	8 1/4	8 1/4	19	19				
B	St1	St1	5	5	8 1/8	8 1/8	19 1/2	19 1/2				
C	St2	St2	5 1/2	5 1/2	8 1/16	8 1/16	19 1/4	19 1/4				
D	St3	St3	5 1/4	5 1/4	16	16	19 1/8	19 1/8				
E	St4	St4	5 1/8	5 1/8	16 1/2	16 1/2	19 1/16	19 1/16				
F	St5	St5	5 1/16	5 1/16	16 1/4	16 1/4						
G	St6	St6	8	8	16 1/8	16 1/8						
H	Ctrl H	Ctrl H	8 1/2	8 1/2	16 1/16	16 1/16						

– Addition of the enzyme conjugate and reaction for 1 hour with a mixture of 5 minutes.

– Washes to remove supernumerary complexes, free cortisol and unbound antibodies.

– Addition of the substrate that will color the complexes in blue (Figure 3).

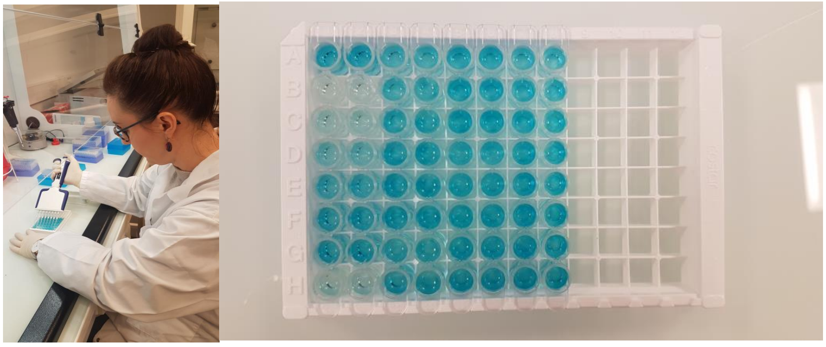

Figure 3. Work under Microbiological Safety Hood (left). Coloring after addition of the substrate (right).

– Reaction for 25 minutes.

– Addition of the stop solution which will transform the blue color into yellow, readable in the visible at 450 nm using a spectrophotometer (Figure 4).

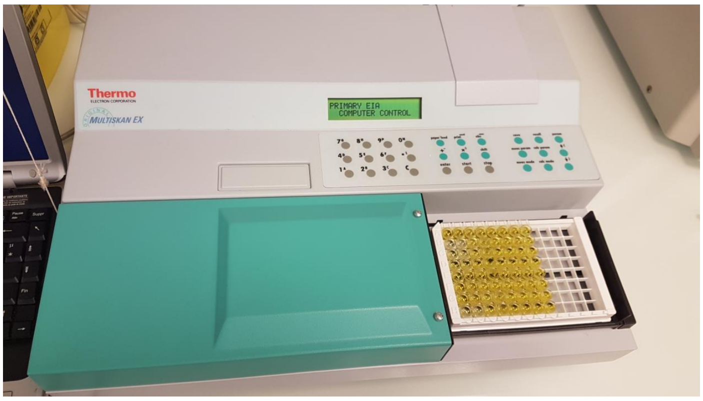

Figure 4. Spectrophotometer and coloring of the plate after addition of the stop solution.

The amount of cortisol assayed in the samples is therefore be inversely proportional to the absorbance measured by the spectrophotometer. We used a logarithmic scale and the 4PL (Four-parameter log-logistic) method to analyze the results. The principle of this competitive sandwich ELISA (Enzyme Linked Immuno Sorbent Assay) is represented in Figure 5.

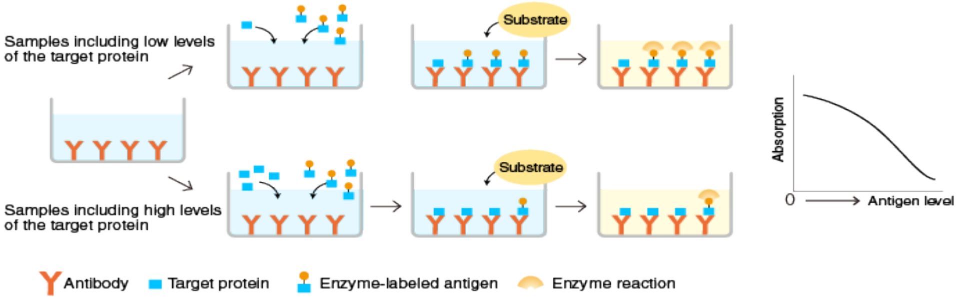

Figure 5. Schematic representation of the competitive sandwich ELISA test.

Results of the parallelism test

The complete results are collated in the form of an Excel file consisting of several tabs (values of absorbance: Table 2).

Table 2: Values of absorbance for the blanck (A1 and A2) standards (B1:G1 and B2:G2), controls (H1, H2, A3, A4), and 4 samples used for the parallelism test (B3:H3, B4:H4, A5:H5, A6:H6, A7:E7, A8:E8)

	1	2	3	4	5	6	7	8	9	10	11	12
A	2.052	2.047	1.351	1.331	1.734	1.747	1.367	1.348				
B	0.169	0.174	1.300	1.390	1.842	1.804	1.550	1.516				
C	0.427	0.377	1.464	1.552	1.833	1.845	1.708	1.656				
D	0.826	0.748	1.688	1.754	1.360	1.376	1.846	1.742				
E	1.388	1.332	1.785	1.964	1.576	1.599	1.865	1.784				
F	1.784	1.757	1.888	1.926	1.804	1.805						
G	1.900	1.907	1.337	1.465	1.852	1.829						
H	0.414	0.400	1.721	1.770	1.931	2.090						

Since the values obtained for the standards fitted well on a logarithmic scale, the 4PL (Four-parameter log-logistic) method could be used to analyze the results (Figure 6). The internal controls were validated with a mean cortisol value of 0.896 µg/dL for the Control High (range: 0.985 ± 0.246 µg/dL) and 0.117µg/dL for the Control Low (range: 0.097 ± 0.024 µg/dL).

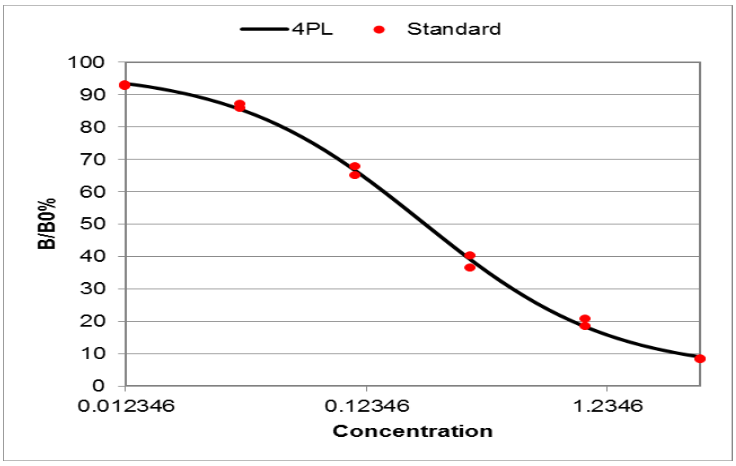

Figure 6. Logarithmic curve of the standards according to the 4PL configuration for the first assay: sinusoidal curve for which extreme values lead to a plateau that is not representative of the actual cortisol values.

The mean percentage of accuracy for the parallelism test is 176.43 ± 20.72 %. The cortisol concentrations of the four diluted samples were not accurate as they differed widely from the expected values calculated by dividing the concentration obtained with no dilution with the coefficients of dilution applied to the samples; Figure 7).

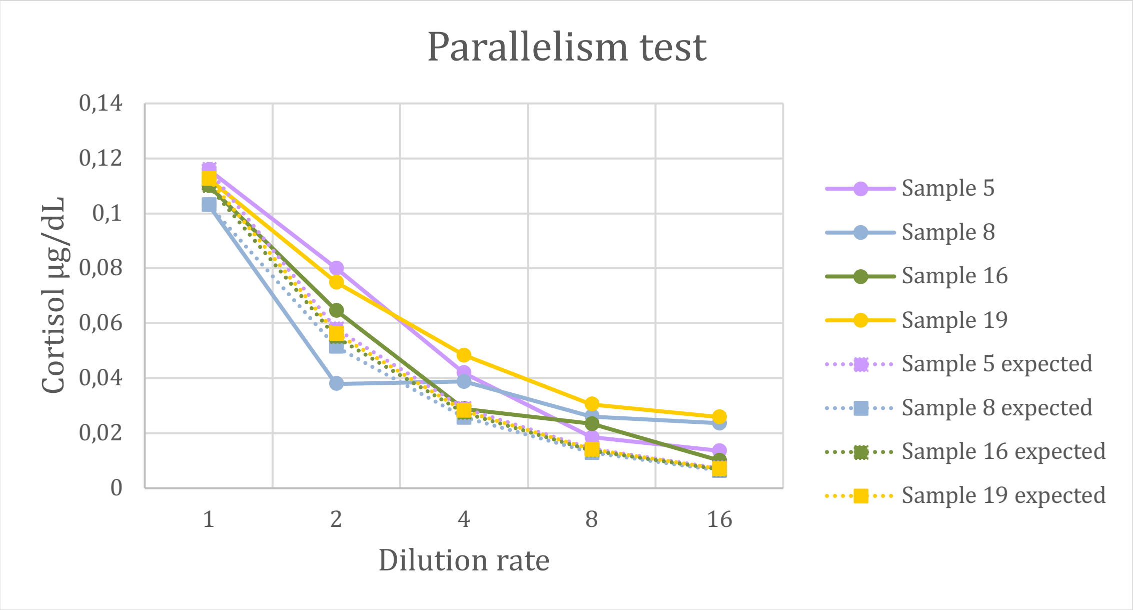

Figure 7: Measured concentrations and expected concentrations of the 4 selected samples, for each dilution rate.

Table 3: Percentages of accuracy for the diluted samples in the parallelism test. Samples with a different background color refer to a subset of four individuals differing by age, sex and group membership. Sample 5 in purple: infant from G3; sample 8 in blue: subadult from G6; sample 16 in green: juvenile from G1; sample 19 in yellow: adult male from G2.Values highlighted in orange: CV is out of 15% range. Values written in red: % accuracy above 150%.

Samples	Dilution	Measured cortisol in µg/dL	Expected cortisol in µg/dL	% accuracy
5	1	0.1159	-	-
8	1	0.1031	-	-
16	1	0.1101	-	-
19	1	0.1126	-	-
5	2	0.0801	0.0580	138.22
8	2	0.0380	0.0516	73.75
16	2	0.0647	0.0551	117.53
19	2	0.0750	0.0563	133.13
5	4	0.0420	0.0290	144.95
8	4	0.0388	0.0258	150.46
16	4	0.0289	0.0275	104.81
19	4	0.0483	0.0282	171.69
5	8	0.0185	0.0145	127.70
8	8	0.0261	0.0129	202.37
16	8	0.0235	0.0138	170.39
19	8	0.0306	0.0141	217.34
5	16	0.0136	0.0072	188.27
8	16	0.0237	0.0064	367.33
16	16	0.0101	0.0069	146.92
19	16	0.0259	0.0070	368.03

We assume that, from the 1/8th dilution, the values were not easily distinguishable because the cortisol concentrations of the four selected samples were already very low (mean ± SE= 0.110 ± 0.003; Table 3), and hence dramatically close to the lowest values that can be detected by the assay when they were diluted at 1/8 and 1/16 (i.e. detectability range = 0.012-3.000 µg/dL).

In addition, two values had to be excluded because intra-assay coefficient of variability CV (i.e. a numerical ratio used to describe the level of variability within a population independently of the absolute values of the observations) was too high. On duplicates, we generally admit a maximum CV of 15% (Table 3).

It should be noted that the values obtained for the four non-diluted samples were almost identical, indicating a lack of variability in cortisol levels, which was unexpected considering that samples from four different individuals varying in age, sex, and dominance had been selected.

Conclusions: Because of the very low measured cortisol levels and a small variation in cortisol levels between the four selected samples, the parallelism test is therefore impossible to fully validate. One may however notice that this test gave some accurate results for the first dilutions steps. Following dosing of the samples had to be carried out without dilution since the values of the pure dosages of the 4 selected samples were in the middle of the range.

b) Second assay: Validation with dose-dependent test and dosing of the remaining samples (January 11, 2019)

The dose-dependent test is another control that assess whether a kit can be used for the determination of cortisol in a new species (here titi monkeys, *P. donacophilus)*. This test evaluates the accuracy of this ELISA by adding a known quantity of a sample (or a mixture of samples) to 3 standard solutions of increasing concentration.

Methods for the dose-dependent test

Here, we took the 4 samples used for the parallelism test and, for each sample, we added 12.5 µL of sample solution to 12.5 µL of standard solutions 2 (Std 2: [cortisol] =[1.000

µg/dL) 3 (Std3: [cortisol] = 0.333 µg/dL) or 4 (Stad4 : [cortisol] = 0.111 µg/dL). We expected the curves obtained for mixtures of standard solutions + samples to be parallel. In addition, we filled 50 remaining wells of the plate to measure the concentrations of the 25 fecal samples of titis that had not been measured yet (samples were dosed in duplicates: 25 samples x 2 wells, Table 4)

Table 4: Plate layout for the blanck (A1 and A2) standards (B1:G1 and B2:G2), controls (H1, H2, A3, A4), 25 samples (B3:H3, B4:H4, A5:H5, A6:H6, A7:H7, A8:H8, A9:D9, A10:D10) and 3 samples used for the dose-dependant test (E9:H9, E10:H10, A11-H11, A12-H12)

	1	2	3	4	5	6	7	8	9	10	11	12
A	Blanc	Blanc	Ctrl L	Ctrl L	10	10	20	20	27	27	8M	8M
B	St1	St1	1	1	11	11	21	21	28	28	8+	8+
C	St2	St2	2	2	12	12	22	22	29	29	16-	16-
D	St3	St3	3	3	13	13			30	30	16M	16M
E	St4	St4	4	4	14	14	23	23	5-	5-	16+	16+
F	St5	St5	6	6	15	15	24	24	5M	5M	19-	19-
G	St6	St6	7	7	17	17	25	25	5+	5+	19M	19M
H	Ctrl H	Ctrl H	9	9	18	18	26	26	8-	8-	19+	19+

The protocol was similar to the one used for the parallelism test, with measure of absorbance used to infer cortisol concentrations (Figure 8).

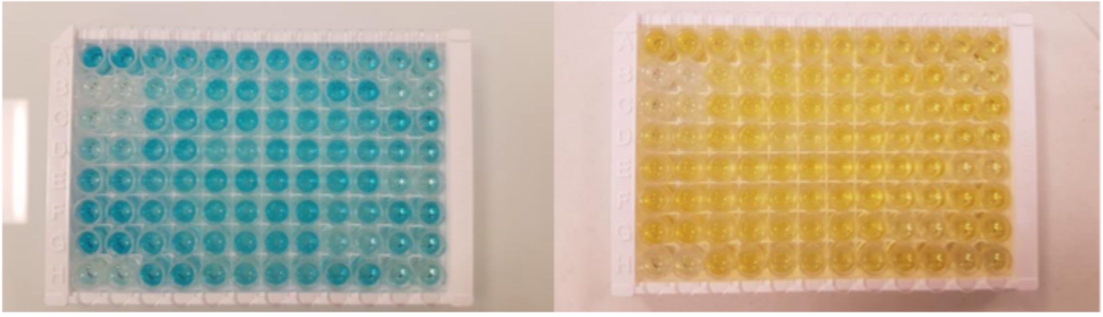

Figure 8. Plates before and after addition of the stop solution.

Results of the assay

The complete results are collated in the form of an Excel file (values of absorbance: Table 5).

Table 5: Values of absorbance for the blanck (A1 and A2) standards (B1:G1 and B2:G2), controls (H1, H2, A3, A4), 25 samples (B3:H3, B4:H4, A5:H5, A6:H6, A7:H7, A8:H8, A9:D9, A10:D10) and 3 samples used for the dose-dependant test (E9:H9, E10:H10, A11-H11, A12-H12)

	1	2	3	4	5	6	7	8	9	10	11	12
A	2.122	2.045	1.392	1.384	1.500	1.505	1.457	1.517	1.220	1.232	1.032	1.089
B	0.203	0.193	0.848	0.890	1.381	1.485	1.323	1.290	1.726	1.701	0.598	0.641
C	0.413	0.391	1.418	1.491	1.489	1.488	1.212	1.264	1.158	1.178	1.430	1.426
D	0.867	0.825	1.464	1.466	0.822	0.836	1.350	1.286	1.178	1.200	1.174	1.081
E	1.412	1.411	1.245	1.294	1.477	1.523	1.465	1.425	1.298	1.370	0.642	0.634
F	1.822	1.787	1.430	1.380	1.325	1.269	1.502	1.530	1.094	1.065	1.455	1.455
G	1.959	1.930	1.320	1.285	1.226	1.281	1.424	1.382	0.601	0.607	1.092	1.113
H	0.403	0.412	1.599	1.611	1.086	1.113	1.198	1.154	1.433	1.397	0.663	0.680

Since the values obtained for the standards fitted well on a logarithmic scale, the 4PL (Four-parameter log-logistic) method could be used to analyze the results (Figure 9).

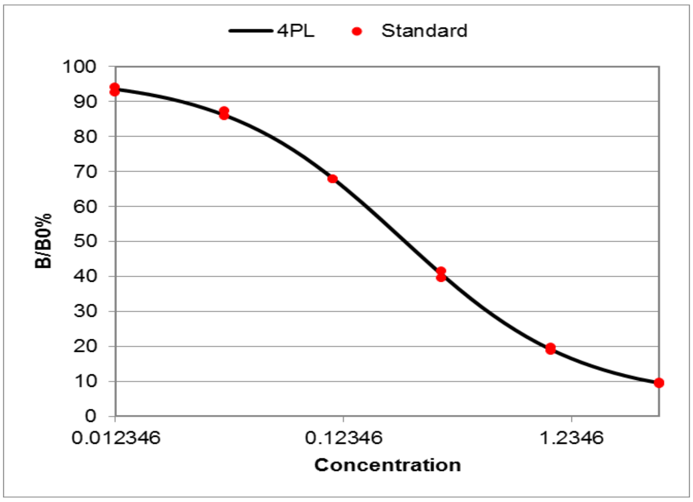

Figure 9. Logarithmic curve of the standards according to the 4PL configuration for the second assay: sinusoidal curve for which extreme values lead to a plateau that is not representative of the actual cortisol values.

The internal controls were validated with a mean cortisol value of 0.973 µg/dL for the Control High (range: 0.985 ± 0.246 µg/dL) and 0.118 µg/dL for the Control Low (range: 0.097 ± 0.024 µg/dL).

For the dose-dependent test, the expected values were calculated by averaging the cortisol value of the pure sample obtained at the time of the parallelism test and the cortisol value of the standard used. The curves obtained for mixtures of standard solutions + samples to

be parallel. (Figure 10). For this dose-dependent test, we found a percentage of accuracy of 91.11 ±2.37 (mean ± SE; Table 6).

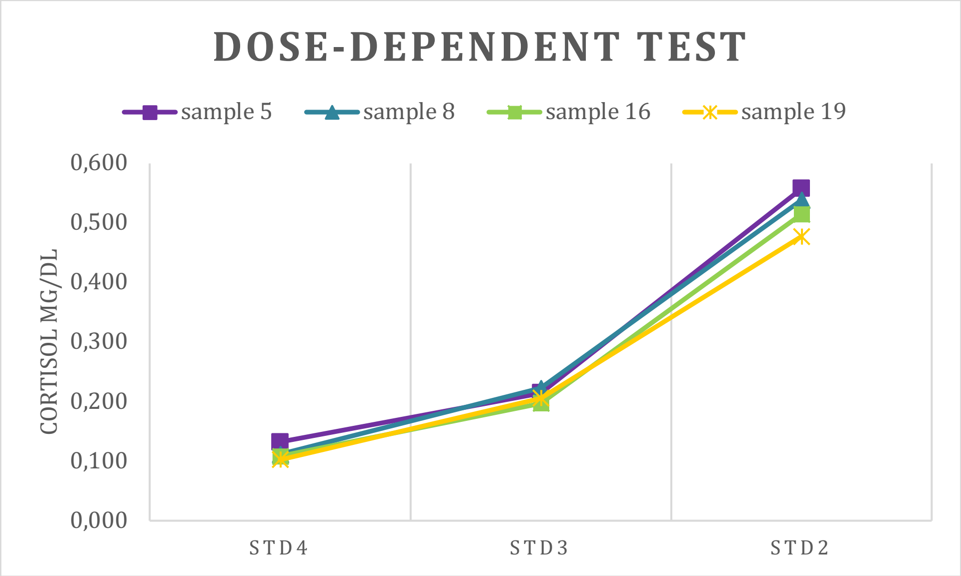

Figure 10: Measured concentrations for the mixtures of standards solutions and each of the 4 selected samples.

Table 6: Percentages of accuracy for the four selected samples in the dose-dependent test (addition of Std4 [cortisol] = 0.111 µg/dL, Std3 [cortisol] = 0.333 µg/dL, or Std2 [cortisol] = 1.000 µg/dL). addition of Samples with a different background color refer to a subset of four individuals differing by age, sex and group membership. Sample 5 in purple: infant from G3; sample 8 in blue: subadult from G6; sample 16 in green: juvenile from G1; sample 19 in yellow: adult male from G2.

Samples	Measured cortisol in µg/dL	Expected cortisol in µg/dL	% accuracy
**5 + Std4**	0.132	0.113	116.615
**5 + Std3**	0.215	0.224	95.567
**5 + Std 2**	0.557	0.558	99.884
**8 + Std4**	0.112	0.107	104.344
**8 + Std3**	0.222	0.218	101.995
**8 + Std 2**	0.538	0.552	97.580
**16 + Std4**	0.109	0.111	98.146
**16 + Std3**	0.197	0.222	88.874
**16+ Std 2**	0.515	0.555	92.712
**19 + Std4**	0.102	0.112	91.503
**19 + Std3**	0.206	0.223	92.280
**19 + Std 2**	0.477	0.556	85.781

The fecal cortisol concentrations for the 30 samples showed little variability in the measured values, with only two samples being above the average multiplied by two (Table 3).

Table 3: Cortisol concentrations for the 30 samples. Values in orange: high values > 2 x mean)

Sample	Date	Group	Individual	CORTISOL µg/dL
1	17/10/2017	G5	Adult female	0.319
2	30/10/2017	G5	Adult male	0.103
3	03/11/2017	G6	Infant	0.100
4	03/11/2017	G6	Juvenile	0.150
5	11/11/2017	G3	Infant	0.116
6	11/11/2017	G3	Juvenile	0.114
7	13/11/2017	G4	Adult female	0.141
8	18/11/2017	G6	Sub adult	0.103
9	22/11/2017	G2	Adult female	0.071
10	23/11/2017	G2	Adult male	0.092
11	28/11/2017	G3	Adult female	0.108
12	28/11/2017	G3	Adult female	0.095
13	02/12/2017	G5	Adult female	0.344
14	02/12/2017	G5	Juvenile	0.092
15	09/12/2017	G1	Adult female	0.142
16	09/12/2017	G1	Juvenile	0.110
17	09/12/2017	G1	Juvenile	0.155
18	12/12/2017	G2	Adult male	0.207
19	12/12/2017	G2	Adult male	0.113
20	19/12/2017	G4	Juvenile	0.095
21	16/01/2018	G2	Juvenile	0.140
22	16/03/2018	G5	Adult female	0.160
23	20/03/2018	G3	Adult male	0.105
24	20/03/2018	G3	Adult male	0.089
25	22/03/2018	G4	Adult male	0.115
26	28/03/2018	G5	Adult female	0.179
27	28/03/2018	G5	Juvenile	0.163
28	05/04/2018	G2	Juvenile	0.051
29	05/04/2018	G1	Adult female	0.182
30	05/04/2018	G1	Adult female	0.175

Conclusions: The dosage of titi monkeys' fecal cortisol with the Salimetrics kit worked perfectly. There is little variability in the measured concentrations, with only two samples above the average multiplied by two.

Literature cited

1. Behringer V. & Deschner T. 2017. Non-invasive monitoring of physiological markers in primates. Hormones and Behavior 91: 3-18.

2. Kalbitzer U. & Heistermann M. 2013. Long-term storage effects in steroid metabolite extracts from baboon (*Papio sp.*) faeces – A comparison of three commonly applied storage methods. Methods in Ecology and Evolution 4 (5): 493-500.

Annex 1: Protocol for the determination of cortisol from faeces using the Salimetrics kit

1) Take out the components of the kit (1h30 before the start of the dosage).

!! The plate is sensitive to humidity, you must leave it in your pocket while it heats up.

2) Dissolution of the samples:

Add 0.5 mL of test buffer to the tubes containing the dry extract (otherwise PBS 1X + 20% ethanol). Shake and then vortex until the pellet is re-dissolved; then put the samples in a water bath (37° C for 30 min). When leaving the water bath, vortex again. If the whole pellet is not re-dissolved, use a micropipette to peel off the rest of the pellet.

Parallelism: make a 2 in 2 dilution with 100 µl of pure solution and 100 µl of test buffer (1/2 to 1/16th).

Test: make the chosen dilution with the parallelism test (test buffer).

Dose-dependent: Add 30 µl of sample diluted with 30 µl of 3 known concentrations of cortisol (high, medium and low).

3) Preparation of the washing solution (1X buffer):

Prepare 150 mL of buffer solution diluted 1:10 (15 mL of buffer solution and 135 mL of distilled water).

!! The diluted solution cannot be stored and should be used on the same day.

!! The value of 150 mL was calculated by counting 4 washes of 96 wells, at the rate of 300 µL per wash.

4) Plate layout:

Determine a plate layout: note the number of associated samples or the range/control number for each well.

5) Well arrangement:

Detach the wells (in strips of 8) and place them on the plate.

6) Preparation of the dilution of the conjugate:

Pipette 24 mL of diluent ("assay diluent") into a tube (minimum 25 mL) to prepare step 9.

7) Filling the wells: Pipette 25 µL of:

- standard (6 wells)
- controls (2 wells)
- samples (80 wells)

!! Ideally, the plate should be ready in less than 20 min.

8) Dilution of the conjugate and arrangement in the wells:

1: 1600 dilution: add 15 µL of conjugate ("cortisol enzyme conjugate") to the tube containing the 24 mL of diluent and shake.

Immediately put 200 µL of this solution in each well.

Note if the wells turn to dark yellow or purple color. If so, use pH strips to determine the pH of the samples.

!! The dosage may be distorted if the pH exceeds 9 or is less than 3.5.

9) Incubation:

Mix (5 min at 500 rpm) and incubate at room temperature for 55 min.

10) Washing:

Wash in 4 times with 1X buffer (prepared in step 3).

With a plate washer or by placing 250 µL of buffer in each well, then inverting the plate over a sink and wiping carefully with paper before putting it back in the place.

11) Addition of the TMB:

Put 200 µL of solution ("TMB substrate solution") in each well.

12) Incubation:

Mix (3 min at 500 rpm) and incubate at room temperature in the dark for 25 min.

13) Stop the reaction:

Add 50 µL of stop solution (“3M stop solution”).

14) Soft mixing and reading:

Mix (3 min at 500 rpm), wipe the plate (the bottom) and read at 450 nm within 10 min following the addition of the stop solution.

!! If the mixture exceeds 600 rpm, liquid may spill.

!! Samples with a value greater than or equal to 3µg/dL (82.7 nmol / L, 30 ng/mL) must be diluted and then reassessed.
