# Supplementary material for: Response of Bolivian gray titi monkeys (Plecturocebus donacophilus) to an anthropogenic noise gradient: behavioral and hormonal correlates
Source: PeerJ. 2020 Nov 20;8:e10417. doi: 10.7717/peerj.10417 (PMC7682439; doi:10.7717/peerj.10417)

**Table S1.1 GLMM analysis with sound pressure (distance: 1–800 m).**

R nlme package using the lme function with age and group as fixed predictors; site nested within track as a random factor. anova is a likelihood ratio test comparing two models. Asterisq shows retained model.

| DV | Model | Fixed and random variables | d.f. | AIC | anova | p-value |
| --- | --- | --- | --- | --- | --- | --- |
| SPL  (log) | m1 | orien x val x h x dist + (1\|track/site) | 43 | -2178.65 | - | - |
|  | m2* | orien + val + h + dist + (1\|track/site) | 11 | -2624.95 | m1,m2 | <.0001 |
|  | m3 | orien + val + h + dist | 9 | -1777.14 | m3,m2 | <.0001 |
|  | m0 | 1, random = ~ 1 \| track/site | 4 | -1476.61 | m2,m0 | <.0001 |

DV: dependent variable; SPL: sound pressure level. d.f.: degree of freedom; AIC: Akaike information criterion

Anova(m1) vAnova(m2)

**
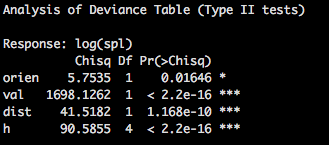

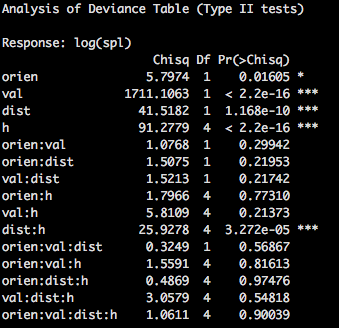
**

**Table S1.2 GLM analysis with sound pressure (distance: 100-800 m).**

Fixed predictors: distance and time-of-day. Asterisq shows retained model.

| DV | Model | Variables | d.f. | AIC | anova | F | p-value |
| --- | --- | --- | --- | --- | --- | --- | --- |
| SPL  (log) | m1 | hour x distance | 350 | -618.3 | - | 17.8 | <0.001 |
|  | m2* | distance x hour | 350 | -618.3 | m1,m2 | 17.8 | <0.001 |
|  | m3 | distance + hour | 354 | -616.7 | m2,m3 | 29.7 | <0.001 |

DV: dependent variable; SPL: sound pressure level; d.f.: degree of freedom; AIC: Akaike information criterion

**
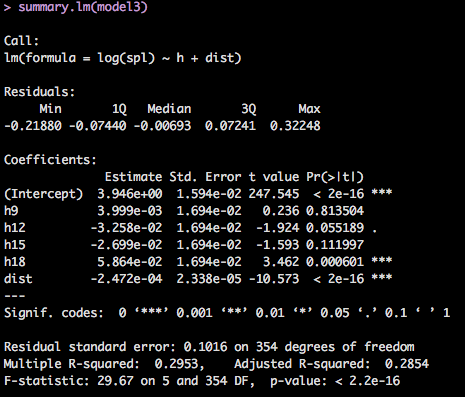

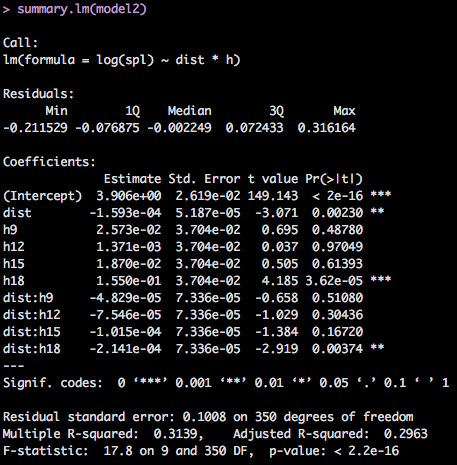
**

**Table S1.3 GLM** **analysis with binomial errors: proportion of time spent moving.**

Fixed predictors: age and group; family=binomial; Asterisqs show retained model.

| DV | Age Class | Model | Variables | d.f. | AIC | z_dist_ | Ov | p-value |
| --- | --- | --- | --- | --- | --- | --- | --- | --- |
| % Moving | Adult | m1 | Distance x sex | 8 | 75.1 | 2.50 | no | 0.013 |
|  |  | m2* | Distance + sex | 9 | 73.4 | 3.06 | no | 0.002 |
|  | Young | m1 | Distance x age | 8 | 93.9 | 1.10 | yes | 0.269 |
|  |  | m2* | Distance + age | 9 | 93.4 | 0.30 | yes | 0.766 |

DV: dependent variable; d.f.: degree of freedom; AIC: Akaike information criterion; Ov: overdispersion.

**
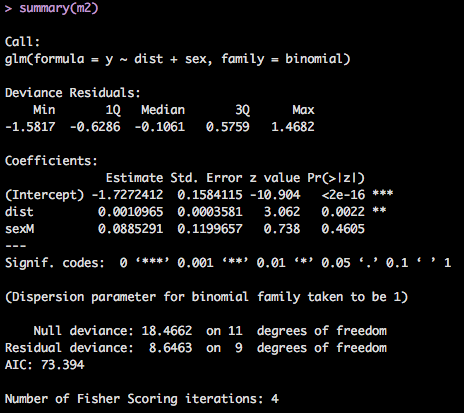
**

**
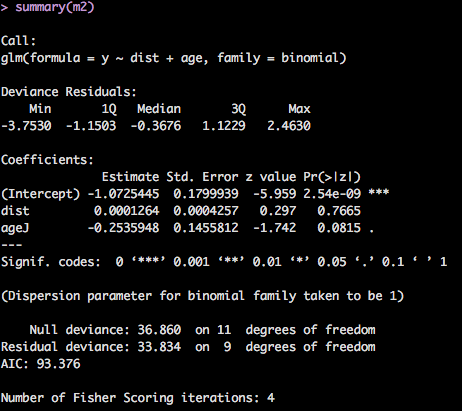
**

**Table S1.4 GLMM analysis with cortisol concentrations**.

Fixed predictors: age and group distance. Random factor: individual nested within group. Asterisq shows retained model.

| DV | Model | Fixed and random variables | d.f. | AIC | anova | p-value |
| --- | --- | --- | --- | --- | --- | --- |
| Cortisol  (1+log.cc) | m1 | Age x distance + (1\|group/id) | 7 | -79.74 | - | - |
|  | m2* | Age + distance + (1\|group/id) | 6 | -99.53 | m1,m2 | <0.0001 |

DV: dependent variable; d.f.: degree of freedom; AIC: Akaike information criterion; cc: concentration;


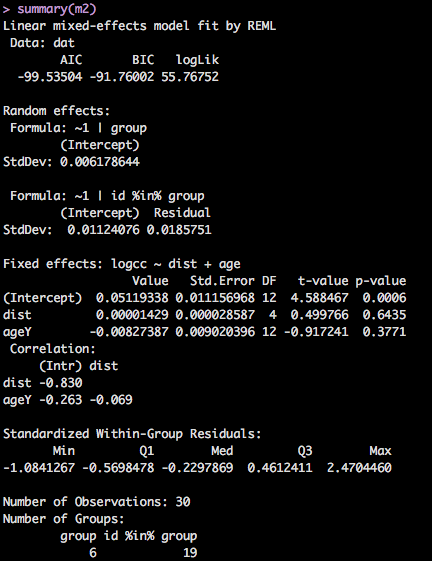

Supplement: Table S1 [file peerj-08-10417-s004.docx]
