# Supplementary material for: Response of Bolivian gray titi monkeys (Plecturocebus donacophilus) to an anthropogenic noise gradient: behavioral and hormonal correlates
Source: PeerJ. 2020 Nov 20;8:e10417. doi: 10.7717/peerj.10417 (PMC7682439; doi:10.7717/peerj.10417)
Supplement: Supplemental Information 6 [file peerj-08-10417-s006.docx]

**SOUND PRESSURE LEVELS: 1-800m**

setwd("~/.")

rm(list=ls(all=TRUE))

dat<-read.csv("data.csv",sep=";")

library(MASS)

library(car)

library(nlme)

library(doBy)

attach(dat)

some(dat)

dat$h<-as.factor(dat$h)

hist(dat$spl)

hist(log10(dat$spl))

shapiro.test(log10(dat$spl))

xtabs(~dist+h,data=dat)

m1 = lme(log(spl) ~ orien + val + dist + h + orien*val*dist*h,

random = ~1|tracknb/samp,

data=dat,

method="REML")

summary(m1)

VarCorr(m1)

Anova(m1)

AIC(m1)

m2 = lme(log(spl) ~ orien + val + dist + h,

random = ~1|tracknb/samp,

data=dat,

method="REML")

summary(m2)

Anova(m2)

AIC(m2)

anova(m1,m2)

----------------------------------------------

**SOUND PRESSURE LEVELS: 100-800m (ANCOVA)**

setwd("~/.")

rm(list=ls(all=TRUE))

dat<-read.csv("data.csv",sep=";")

library(doBy)

library(seewave)

dat$h<-as.factor(dat$h)

attach(dat)

model1 <- lm(log(spl)~h*dist)

summary.lm(model1)

model2 <- lm(log(spl)~dist*h)

summary.lm(model2)

model3 <- lm(log(spl)~h+dist)

summary.lm(model3)

anova(model1,model2)

summaryBy(spl~h, data=dat, FUN=c(meandB,sddB),level="SPL")

summaryBy(spl~dist, data=dat, FUN=c(meandB,sddB),level="SPL")

summaryBy(spl~samp, data=dat, FUN=c(meandB,sddB),level="SPL")

summaryBy(spl~h*dist, data=dat, FUN=c(meandB,sddB),level="SPL")

------------------------------------------------

**#Testing the regression line:NIGHT**

distance<-c(38,54,54,77,119,162,204,299,301,308,310,366,403,407,465,468,483,484,518,525,576,586,606,639,691,740,761)

night<-c(1756.5472, 1641.0846, 1047.1251, 1074.5957, 1201.7998, 759.6694, 549.5439, 779.1753, 635.1295, 494.4959, 592.6221, 661.1106, 540.6447, 488.5342, 576.1216, 675.5415, 1980.3729, 875.5632, 852.1576, 551.0423, 673.4308, 450.5994, 608.1347, 559.3893, 355.7934, 352.1076, 359.1858)

X<-distance

Y<-log(night)

shapiro.test(Y)

SSX <- sum(X^2)-sum(X)^2/length(X)

SSY <- sum(Y^2)-sum(Y)^2/length(Y) # total variation SSY

SSXY <- sum(X*Y)-sum(X)*sum(Y)/length(X)

SSXY

SSXY/SSX

SSR<-(SSXY/SSX)*SSXY

SSE<-SSY-SSR

n = 27

SSE/(n-2)

(SSE/(n-2))/SSR

r2<-SSR/SSY

r<-sqrt(r2)

model <- lm(Y~X)

summary(model)

summary.aov(model)

par(mfrow=c(2,2))

plot(model)

influence.measures(model)

summary(model)

r

**#Testing the regression line:DAY**

distance<-c(38,54,54,77,119,162,204,299,301,308,310,366,403,407,465,468,483,484,518,525,576,586,606,639,691,740,761)

day<-c(2546.6598, 3157.8906, 1506.8941, 2376.6596, 2253.6587, 915.8618, 1257.6977, 1218.9797, 892.4882, 923.6882, 755.7556, 1242.7899, 416.6460, 853.4127, 489.1487, 1196.1947, 798.3348, 1948.0294, 1018.9043, 734.6583, 755.9845, 576.3732, 874.2035, 668.8665, 350.8975, 434.8638, 440.4011)

X<-distance

Y<-log(day)

shapiro.test(Y)

SSX <- sum(X^2)-sum(X)^2/length(X)

SSY <- sum(Y^2)-sum(Y)^2/length(Y) # total variation SSY

SSXY <- sum(X*Y)-sum(X)*sum(Y)/length(X)

SSXY

SSXY/SSX

SSR<-(SSXY/SSX)*SSXY

SSE<-SSY-SSR

n = 27

SSE/(n-2)

(SSE/(n-2))/SSR

r2<-SSR/SSY

r<-sqrt(r2)

model <- lm(Y~X)

summary(model)

summary.aov(model)

par(mfrow=c(2,2))

plot(model)

influence.measures(model)

summary(model)

r

------------------------------------------------------------------------------------

**GROUP ACTIVITY BUDGETS**

B = matrix(c(153,81,113,28,16,211,99,97,78,18,153,109,91,62,41,362,159,116,69,29,149,105,92,69,31,125,100,80,32,29),nrow=5,ncol=6)

B<-t(B)

a<-data.frame(B)

library(grid)

all_p<-data.frame()

for (i in 6:1) {

p<-round(prop.table(a[i,1:5])*100,2)

all_p<-rbind(p,all_p)

}

all_p

colnames(B)=c("re","mo","fo","ob","so")

rownames(B)=c("G1","G2","G3","G4","G5","G6")

colnames(all_p)=c("re","mo","fo","ob","so")

rownames(all_p)=c("G1","G2","G3","G4","G5","G6")

library(psych)

describe(all_p)

obs<-as.matrix(B)

#Post-hoc pairwise chi-square tests

library(rcompanion)

pairwiseNominalIndependence(obs,

fisher = FALSE,

gtest = FALSE,

chisq = TRUE,

method = "fdr")

#Post-hoc pairwise chi-square tests with pairwise.table

FUN = function(i,j){

chisq.test(matrix(c(obs[i,1], obs[i,2],

obs[j,1], obs[j,2]),

nrow=2,

byrow=TRUE))$ p.value

}

pairwise.table(FUN,

rownames(obs),

p.adjust.method="bonferroni")

chisq <- chisq.test(B)

esp<-round(chisq$expected,2)

chiporcelda<-(obs-esp)^2/esp

chiporcelda

------------------------------------------------

**TIME SPENT IN EACH FOREST STRATUM: Comparing S1 vs. S2+S3**

B = matrix(c(261,122,364,139,251,202,468,257,333,107,270,84),nrow=2,ncol=6)

B<-t(B)

a<-data.frame(B)

library(grid)

all_p<-data.frame()

for (i in 6:1) {

p<-round(prop.table(a[i,1:2])*100,2)

all_p<-rbind(p,all_p)

}

colnames(B)=c("s1","s2")

rownames(B)=c("G1","G2","G3","G4","G5","G6")

colnames(all_p)=c("s1","s2")

rownames(all_p)=c("G1","G2","G3","G4","G5","G6")

all_p

library(psych)

describe(all_p)

wilcox.test(all_p$s2,all_p$s1, paired=T)

obs<-as.matrix(B)

#Post-hoc pairwise chi-square tests

library(rcompanion)

pairwiseNominalIndependence(obs,

fisher = FALSE,

gtest = FALSE,

chisq = TRUE,

method = "fdr")

#Post-hoc pairwise chi-square tests with pairwise.table

FUN = function(i,j){

chisq.test(matrix(c(obs[i,1], obs[i,2],

obs[j,1], obs[j,2]),

nrow=2,

byrow=TRUE))$ p.value

}

pairwise.table(FUN,

rownames(obs),

p.adjust.method="none")

chisq <- chisq.test(B)

esp<-round(chisq$expected,2)

chiporcelda<-(obs-esp)^2/esp

chiporcelda

--------------------------------------------------

**ANCOVA: Regression with binomial errors**

#distance=continuous, sex=categorical

for<-c(46,20,6,43,28,17,21,28,31,36,25,21)

rest<-c(71,47,46,123,28,20,53,60,39,113,53,40)

mov<-c(30,20,27,53,31,21,14,29,13,65,29,26)

obs<-c(8,19,18,28,22,5,5,29,9,28,20,10)

soc<-c(3,6,14,9,13,6,11,2,5,9,7,9)

tot<-c(158,112,111,256,122,69,104,148,97,251,134,106)

sex<-c(rep("M",6),rep("F",6))

n<-cbind(tot,mov)

class(n)

n<-data.frame(n)

colnames(n)<-c("tot","mov")

n$dist<-c(66,184,281,420,514,592,66,184,281,420,514,592)

n$p <- round(mov/tot,4)

n$dif<-tot-mov

attach(n)

par(mfrow=c(1,2))

plot(dist,p,ylab="Proportion moving")

plot(log(dist),p,ylab="Proportion moving")

y <- cbind(mov,dif)

pf<- round(mov/tot,4)

pfc <- split(pf,sex)

dc <- split(dist,sex)

plot(dist,pf,type="n",ylab="Proportion moving")

points(jitter(dc[[1]]),jitter(pfc[[1]]),pch=21,bg="red")

points(jitter(dc[[2]]),jitter(pfc[[2]]),pch=22,bg="blue")

m1 <- glm(y~dist*sex,binomial)

summary(m1)

m2 <- glm(y~dist+sex,binomial)

summary(m2)

--------------------------------------------------

**Logistic regression with binomial errors**

#adult male: % moving

mov<-c(30,20,27,53,31,21)

tot<-c(158,112,111,256,122,69)

n<-cbind(tot,mov)

class(n)

n<-data.frame(n)

colnames(n)<-c("tot","mov")

n$dist<-c(66,184,281,420,514,592)

n$p <- mov/tot

n$dif<-tot-mov

#n$dif<-c(128,92,84,203,91,48)

attach(n)

par(mfrow=c(1,2))

plot(dist,p,ylab="Proportion moving")

plot(log(dist),p,ylab="Proportion moving")

y <- cbind(dif,mov)

m1 <- glm(y~dist,binomial)

summary(m1)

m2 <- glm(y~log(dist),binomial)

summary(m2)

------------------------------------------------------

**Logistic regression with binomial errors**

#adult female: % moving

mov<-c(14,29,13,65,29,26)

tot<-c(104,148,97,251,134,106)

n<-cbind(tot,mov)

class(n)

n<-data.frame(n)

colnames(n)<-c("tot","mov")

n$dist<-c(66,184,281,420,514,592)

n$p <- round(mov/tot,4)

n$dif<-tot-mov

#n$dif<-c(90,119,84,186,105,80)

attach(n)

par(mfrow=c(1,2))

plot(dist,p,ylab="Proportion moving")

plot(log(dist),p,ylab="Proportion moving")

y <- cbind(mov,dif)

m1 <- glm(y~dist,binomial)

m2 <- glm(y~log(dist),binomial)

summary(m2)

-----------------------------------------------------

**SOCIAL PROXIMITY:** **tight** **social dyads**

Bs = matrix(c(72,0,28,12,0,32,34,0,0,2,18,0,26,8,0,15,5,0,0,0, 67,25,11,63,23,3,41,10,22,15,8,6,12,4,5,5,2,1,2,1, 63,0,51,53,0,27,48,0,0,49,11,0,15,5,0,9,4,0,0,2, 186,0,79,21,0,65,25,0,0,16,52,0,12,0,0,20,7,0,0,1, 62,0,50,38,0,28,27,0,0,32,11,0,8,3,0,14,2,0,0,1, 48,16,19,26,0,12,23,10,4,20,10,1,8,6,2,3,2,0,3,4),nrow=10,ncol=12)

Bs<-t(Bs)

a<-data.frame(Bs)

library(grid)

all_p<-data.frame()

for (i in 12:1) {

p<-round(prop.table(a[i,1:10])*100,2)

all_p<-rbind(p,all_p)

}

colnames(Bs)=c("FM","FS","FJ","FI","MS","MJ","MI","SJ","SI","JI")

rownames(Bs)=c("G1","g1","G2","g2","G3","g3","G4","g4","G5","g5","G6","g6")

colnames(all_p)=c("FM","FS","FJ","FI","MS","MJ","MI","SJ","SI","JI")

rownames(all_p)=c("G1","g1","G2","g2","G3","g3","G4","g4","G5","g5","G6","g6")

all_p # proportions

#Tight social dyads

TOT<-sum(Bs[1,])+sum(Bs[3,])+sum(Bs[5,])+sum(Bs[7,])+sum(Bs[9,])+sum(Bs[11,])

#Social-dyads

x1<-c(40.00,23.93,21.65,47.45,26.16,26.97)#FM

mean(x1), sd(x1)

x2<-c(15.56,3.93,17.53,20.15,21.10,10.67)#FJ

mean(x1), sd(x1)

x3<-c(17.78,1.07,9.28,16.58,11.81,6.74) #MJ

mean(x1), sd(x1)

x4<-c(6.67,22.50,18.21,5.36,16.03,14.61) #FI

mean(x1), sd(x1)

x5<-c(18.89,14.64,16.49,6.38,11.39,12.92)#MI

mean(x1), sd(x1)

x6<-c(1.11,5.36,16.84,4.08,13.5,11.24)#IJ

mean(x1), sd(x1)

x7<-c(8.93,8.99)#SF

mean(x1), sd(x1)

x8<-c(8.21,0.00)#SM

mean(x1), sd(x1)

x9<-c(3.57,5.62)#SJ

mean(x1), sd(x1)

x10<-c(7.86,2.25)#SI

mean(x1), sd(x1)

**----------------------------**-----------------------------------------------------

**Effect of mannequin on titis’ alarm response**

#G-test for goodness-of-fit

observed = c( 0, 1, 150, 62, 59, 71)

theoretical = c(1/6, 1/6, 1/6, 1/6, 1/6, 1/6)

library(DescTools)

GTest(x = observed,

p = theoretical,

correct="none")

#Check expected counts

Test = GTest(x = observed,

p = theoretical,

correct="none")

Test$expected

#area anthropic disturbance

observed = c(1, 212, 130)

theoretical = c(1/3, 1/3, 1/3)

library(DescTools)

GTest(x = observed,

p = theoretical,

correct = "none")

#Check expected counts

Test = GTest(x = observed,

p = theoretical,

correct = "none")

Test$expected

#Post-hoc analysis:standardized residuals

observed = c(0, 1, 150, 62, 59, 71)

theoretical = c(1/6, 1/6, 1/6, 1/6, 1/6, 1/6)

chisq.test(x = observed, p = theoretical)$stdres

#area anthropic disturbance

observed = c(1,212,130)

theoretical = c(1/3, 1/3, 1/3)

chisq.test(x = observed, p = theoretical)$stdres

-------------------------------------------------

**Cortisol concentration: effect of age and distance**

setwd("~/.")

rm(list=ls(all=TRUE))

dat<-read.csv("data.csv", sep=";")

dat$logcc<-log10(1+dat$cc)

library(nlme)

m1 = lme(logcc ~ dist*age, #group distance = 66,184,281,420,514,592 (entered as a covariate)

random = ~1|group/id,

data=dat,

method="REML")

print(m1, correlation=FALSE)

m2 = lme(logcc ~ dist+age,

random = ~1|group/id,

data=dat,

method="REML")

print(m2, correlation=FALSE)

anova(m1,m2)

anova(m1,m2, test="F")

library(car)

Anova(m2)

---------------------------------------------------

**Timing sample collection: morning vs. afternoon**

setwd("~/.")

rm(list=ls(all=TRUE))

dat<-read.csv("data.csv", sep=";")

dat<-dat[c(-23,-11,-10,-7),]

manana<-subset(dat,period=="manana")

tarde<-subset(dat,period=="tarde")

boxplot(manana$cc, tarde$cc)

man<-manana$cc

tar<-tarde$cc

#Wilcoxon rank sum test = Mann Whitney U test

wilcox.test(man,tar)

--------------------------------------------------

**Pearson Regression analysis: CORT vs. RMS**

rms<-c(1264.4779, 830.6948, 711.0812, 1067.4894, 653.6029, 440.2810)

med<-c(0.08211225, 0.05038335, 0.04770676, 0.05742501, 0.04239391, 0.02919075)

cc<-c(0.1494084, 0.1043912, 0.1070175, 0.1168200, 0.1602217, 0.1177333)

#Pearson Regression analysis: CORT vs. RMS

X<-cc

Y<-rms

Y<-log(Y)

shapiro.test(X)

shapiro.test(Y)

model <- lm(Y~X)

summary(model)

summary.aov(model)

par(mfrow=c(2,2))

plot(model)

influence.measures(model)

#Pearson Regression analysis: CORT vs. M

Y<-med

Y<-log(Y)

shapiro.test(X)

shapiro.test(Y)

model <- lm(Y~X)

summary(model)

summary.aov(model)

par(mfrow=c(2,2))

plot(model)

influence.measures(model)

------------------------------------------------------
